# Neurostimulation Combined With Cognitive Intervention in Alzheimer’s Disease (NeuroAD): Study Protocol of Double-Blind, Randomized, Factorial Clinical Trial

**DOI:** 10.3389/fnagi.2018.00334

**Published:** 2018-11-02

**Authors:** Suellen Marinho Andrade, Eliane Araújo de Oliveira, Nelson Torro Alves, Ana Cristina Gomes dos Santos, Camila Teresa Ponce Leon de Mendonça, Danielle Dorand Amorim Sampaio, Edyllaine Elidy Querino Cavalcante da Silva, Égina Karoline Gonçalves da Fonsêca, Evelyn Thais de Almeida Rodrigues, Gabriela Nayara Siqueira de Lima, Jamerson Carvalho, Jessyca Alves Silvestre da Silva, Manuella Toledo, Marine Raquel Diniz da Rosa, Marcia Queiroz de Carvalho Gomes, Melquisedek Monteiro de Oliveira, Moema Teixeira Maia Lemos, Nágylla Gomes Lima, Penha Inácio, Petra Maria da Cruz Ribeiro e Rodrigues, Rayssa Gabriela Dantas Ferreira, Renata Cavalcante, Renata Emanuela Lyra de Brito Aranha, Regina Neves, Rodrigo Marmo da Costa e Souza, Thainá Magalhães Portugal, Wanessa Kallyne Nascimento Martins, Vivian Pontes, Thiago Monteiro de Paiva Fernandes, Israel Contador, Bernardino Fernández-Calvo

**Affiliations:** ^1^Department of Physiotherapy, The Federal University of Paraíba, João Pessoa, Brazil; ^2^Department of Psychology, The Federal University of Paraíba, João Pessoa, Brazil; ^3^Department of Occupational Therapy, The Federal University of Paraíba, João Pessoa, Brazil; ^4^Department of Internal Medicine, The Federal University of Paraíba, João Pessoa, Brazil; ^5^Department of Speech-Language Pathology and Audiology, The Federal University of Paraíba, João Pessoa, Brazil; ^6^Brazilian Alzheimer’s Association, João Pessoa, Brazil; ^7^Department of Basic Psychology, Psychobiology and Methodology of Behavioral Science, University of Salamanca, Salamanca, Spain

**Keywords:** clinical trial, dementia, neuropsychology, non-pharmacological approaches, transcranial direct current stimulation, neuromodulation

## Abstract

Despite advances in the treatment of Alzheimer’s disease (AD), there is currently no prospect of a cure, and evidence shows that multifactorial interventions can benefit patients. A promising therapeutic alternative is the use of transcranial direct current stimulation (tDCS) simultaneously with cognitive intervention. The combination of these non-pharmacological techniques is apparently a safe and accessible approach. This study protocol aims to compare the efficacy of tDCS and cognitive intervention in a double-blind, randomized and factorial clinical trial. One hundred participants diagnosed with mild-stage AD will be randomized to receive both tDCS and cognitive intervention, tDCS, cognitive intervention, or placebo. The treatment will last 8 weeks, with a 12-month follow-up. The primary outcome will be the improvement of global cognitive functions, evaluated by the AD Assessment Scale, cognitive subscale (ADAS-Cog). The secondary outcomes will include measures of functional, affective, and behavioral components, as well as a neurophysiological marker (Brain-derived neurotrophic factor, BDNF). This study will enable us to assess, both in the short and long term, whether tDCS is more effective than the placebo and to examine the effects of combined therapy (tDCS and cognitive intervention) and isolated treatments (tDCS vs. cognitive intervention) on patients with AD.

**Clinical Trial Registration**: www.ClinicalTrials.gov, identifier NCT02772185—May 5, 2016.

## Introduction

Alzheimer’s disease (AD) is characterized by a progressive deterioration of cognitive functions, impacting the autonomy of patients in performing their activities of daily living (ADLs; Buschert et al., 2010). Pharmacological approaches only provide moderate symptom control, provisionally improving cognition or slowing down cognitive decline (Massoud and Gauthier, 2010). In this context, the earlier the diagnosis of probable AD is performed, the better the patient prognosis will be as cognitive intervention can be applied in the early stages of the disease (Fernández-Calvo et al., 2015; Kawashima et al., 2015). For example, memory, executive functions and attention may be relatively improved when adequate support is provided (García-Alberca, 2015). However, intervention specificity, best candidate selection for different types of protocols, and whether benefits can transfer to untrained cognitive domains are still controversial topics (Alves et al., 2013; Fernández-Calvo et al., 2015).

Thus, multimodal intervention makes it possible to prevent (Olanrewaju et al., 2015) or delay the progression caused by the disease (Olazarán et al., 2010). Currently, non-invasive neurostimulation techniques such as transcranial direct current stimulation (tDCS) have been used to delay the cognitive deficits and to decrease the functional impairment of patients with dementia (Andrade et al., 2016; André et al., 2016; Gonsalvez et al., 2017). tDCS is a low-cost, portable and safe tool that induces plasticity via electrical stimulation using a low-intensity current and a continuous flow through the brain. This pattern of electricity modulates cortical activity without directly acting on neurons, which decreases the risk of serious adverse effects (Boggio et al., 2011).

The neurobiological basis of AD recovery after neurostimulation is the ability of tDCS to induce long-term synaptic changes. The effects perceived during stimulation are apparently associated with changes in the subliminal membrane resting levels that modulate the input-output curve. Conversely, prolonged effects would act on GABAergic activity, the glutamatergic system, long-term potentiation (LTP) and long-term depression (LTD) mechanisms related to plasticity (Yu et al., 2015). Studies in mice with a similar disease to AD show tDCS effects on several of these factors, also including axonal activation and alteration in the cellular transmembrane potential, and consequently neurotransmitter balance (Ruohonen and Karhu, 2012).

In a critical review of the use of tDCS combined with cognitive rehabilitation, Cappon et al. (2016) concluded that many studies failed to assess whether improvement in a specific task transfers to ADLs in AD treatment. The authors indicate it is imperative to discriminate between increased performance on a specific cognitive task and recovery in more general ADLs demanding that cognitive function. This suggests the importance of including other cognitive domains in both the evaluation and the treatment protocol, such as memory and executive function. Consistent results reported by systematic reviews and meta-analyses have shown that overall cognitive stimulation, a cognitive intervention based on a range of different cognitive activities (Clare and Woods, 2004), promotes better cognitive and neuropsychiatric benefits in patients with AD than cognitive training of specific domains (e.g., memory; Buschert et al., 2010; Spector et al., 2012; Woods et al., 2012; Bahar-Fuchs et al., 2013; Huntley et al., 2015).

Nevertheless, some studies only evaluated the effect of different forms of neurostimulation without associating neurostimulation with cognitive intervention protocols (Boggio et al., 2009, 2011; Bystad et al., 2016b). To date, three studies have evaluated the combined use of tDCS and cognitive rehabilitation (Cotelli et al., 2014; Penolazzi et al., 2015; Andrade et al., 2016). A common limitation to these studies is the use of a brief protocol of tDCS (only 10 sessions), without long-term follow-up of participants (longer than 6 months). Because AD is a progressive disease, monitoring participants for long periods of time is crucial to assess whether the therapeutic effect is sustained to enable the tested protocol to be transferred to clinical practice. However, a thorough search of the relevant literature yielded no articles analyzing tDCS efficacy associated with cognitive intervention in producing physiological and behavioral changes in patients over time (12 months).

Additionally, the current application moment, meaning whether it is applied before, during, or after cognitive intervention, may also cause variability in the results (Cappon et al., 2016). In a systematic review and meta-analysis assessing the effects of non-invasive neurostimulation on healthy elderly individuals and patients with AD, the authors observed that “the optimal timing for administration of tDCS” varies according to the physiological and pathological aging (Hsu et al., 2015). The use of neurostimulation before task execution can produce robust effects on healthy elderly individuals. Conversely, AD group shows more pronounced effects for studies that apply the stimulation during task execution compared to studies that uses the stimulation before task execution.

Another key point is that most studies involving tDCS in AD typically assess neurostimulation effects on only one cortical area. This aspect is worrying because the pathological characterization of dementia differs from other neurological diseases, wherein only one brain region should be modulated such as stroke or depression (Iannone et al., 2016; Ilić et al., 2016). In AD, the clinical symptoms are not restricted to memory loss, suggesting the involvement of more complex neural networks (Lesuis et al., 2016; Ossenkoppele et al., 2016). Thus, choosing only the frontal or temporal region could limit the scope of treatment and result in the absence of cognitive gains, as found by Bystad et al. (2016a) who observed no improvement in the cognitive domains of patients when applying tDCS over a short period (10 days) exclusively on the left temporal cortex. Thus, it is important that the therapeutic protocol of tDCS in AD should encompass another cortical locus.

Related to this, other factors must be analyzed such as the stimulation duration. Although the standard tDCS protocol only involves application in one cortical area per session of 20 min (Nitsche et al., 2008), some authors also reported that 10 min of tDCS application in one (Drummond et al., 2017) or two cortical regions, one after the other (frontal and parietal cortex; Jones et al., 2015), is sufficient to promote neuromodulatory effects. In addition, Fricke et al. (2011) prove that the use of repeated applications of tDCS generates long-lasting effects.

Although there are several studies involving non-invasive neurostimulation and cognitive training in the treatment of AD, the cognitive and functional effects remain unclear. Recent reviews suggest larger samples to investigate the synergistic effects of these two therapies, with control of confounding variables and with realistic parameters, aimed at clinical practice (Hsu et al., 2015; Zhao et al., 2017).

Accordingly, we propose that stimulating different brain regions specifically related to the neuropathology of AD along with repeated applications of 10-min tDCS in each session may be an option to achieve short- and long-term effects. However, at this time we have only just drawn up a study protocol and these hypotheses can only be tested in the main clinical trial that we aim to perform. In addition, no reports of factorial randomized clinical trials using with tDCS and cognitive intervention stimulating different cortical regions with a 10-min interval in each, and with follow-up for AD-related outcomes have been found to date.

In this way, we designed the Neurostimulation combined with Cognitive Intervention in AD (NeuroAD) clinical study, a phase II/III trial, to address the safety and efficacy of tDCS and cognitive intervention in the treatment of AD. Based on the aforementioned data, which suggest beneficial effects of both cognitive intervention and tDCS on patients with AD, we hypothesize that combining both treatments modulates cortical activity and improves the long-term clinical symptoms of patients, manifested as increased cognition and functional capacity and reduced affective and behavioral changes.

## Materials and Equipment

### Overview

NeuroAD is a factorial, placebo-controlled, double-blind and randomized clinical trial in which 100 patients at the mild stage of AD are randomly assigned to four groups: 1—active tDCS and active cognitive intervention; 2—sham tDCS and active cognitive intervention; 3—active tDCS and placebo cognitive intervention; and 4—sham tDCS and placebo cognitive intervention. All of the patients will be subjected to tDCS and cognitive intervention applied for 24 workdays. At the end of the trial, patients who received active tDCS and achieved clinical response will be invited to maintain tDCS bimonthly for 12 months, as part of a long-term study of tDCS for AD; those who did receive sham tDCS and did not respond will be offered 24 open-label daily sessions of tDCS (crossover study); finally, those who showed response with cognitive intervention (real or placebo) and sham tDCS will be referred to other neuropsychological centers specialized in treating dementias in our Institution.

### Randomization and Blinding

The participants will be randomized into 1:1:1:1 blocks using the random number generator of an open-access randomization software program^1^. This sequence will be remotely performed by a blind researcher not involved in other research procedures. After the randomization process, a blinded staff member will perform patient allocation between the groups. Hidden allocation will be performed with closed, sealed and sequentially numbered envelopes. The participants will be identified by codes and will also be blinded; that is, they will ignore the arm of the study to which they will be allocated. The evaluation and treatment will be performed by independent researchers, who will identify the patients by their respective codes and will be blinded to the other research procedures. Data analysis will be performed by a professional not involved in any stage of patient recruitment, screening, assessment, or intervention. The effectiveness of the masking will be assessed after completing the treatment by interviewing the raters, who will be asked what type of treatment (active or placebo) they believe that the patient received. The relatives of participants will also be blinded and asked, at the end of the treatment, to which group they believe that the patient was allocated. The patients will also be asked questioned about which group they believe have been allocated. We decided to assess the effectiveness of the blinding by asking the relatives and/or caregivers of the patients because the patients have mild AD.

### Attrition and Adherence

Attrition will be considered under the following conditions: (a) two consecutive or three alternating absences from treatment sessions in a one-month period; (b) the inability to complete the post-test and follow-up; and (c) the development of any disabling condition preventing the participant’s participation in the study. Regarding adherence strategies, sessions missed until the established limit will be reset the following week. Flexible therapy hours will also be offered, and the relatives of the patients will be directly contacted by telephone to confirm the dates of evaluation, thereby reinforcing treatment adherence (Brunoni et al., 2011). Additional measures to avoid dropouts will also be applied, including periodic assessments of treatment satisfaction, discussion of difficulties in continuing treatment (for example, logistics of the trips to the laboratory) and attempts to resolve and avoid possible problems that may affect adherence to and continued participation in the study.

### Safety

To investigate adverse effects, the participants will be asked at the end of each session whether they experienced effects such as “tingling”, “burning”, “headache”, “somnolence”, among others and subsequently asked the intensity of this sensation (1—none, 2—mild, 3—moderate, 4—strong) and whether this effect could be related to stimulation using a Likert scale ranging from 1 (unrelated) to 5 (strongly related). Although tDCS is considered a safe technique that has been applied to several neurological and psychiatric disorders for years (Nardone et al., 2015), if the patient exhibits any severe impairment or discomfort, treatment will be discontinued, and medical assistance and physical and psychological therapy will be provided to control potential problems and promote recovery.

### Participants

The evaluation and intervention procedures applied to the participants will be performed by trained professionals with experience in AD patient management. The team will consist of neuropsychologists, geriatricians, neurologists, occupational and speech therapists, biomedical and physiotherapists.

#### Inclusion Criteria

The participants will be included according to the following eligibility criteria: (a) being 50–90 years of age; (b) having a diagnosis of dementia [criteria of the Diagnostic and Statistical Manual of Mental Disorders—Fourth Edition (Text Revision, DSM-IV-TR; American Psychiatric Association, 2000)] and of probable AD according to the National Institute of Neurology and Communication Disorder and Stroke-The AD and Related Disorders Association Criteria (NINCDS-ADRDA; McKhann et al., 2011); (c) having a score = 1 in the Clinical Dementia Rating (CDR; Morris, 1993); (d) a score higher than 18 points in the Mini-Mental State Exam (MMSE; Folstein et al., 1975); (e) using acetylcholinesterase inhibitors (AChEI) and/or memantine for at least 3 months prior to screening; and (f) not having received regular cognitive intervention in the 3 months prior to the start of this clinical trial.

#### Exclusion Criteria

Participants (a) with unstable medical conditions, serious metabolic and/or cardiac diseases, alcoholism, focal neurological disorders (e.g., epilepsy, stroke, brain injury or tumor) and associated psychiatric disorders; or (b) using hypnotics and benzodiazepines up to 2 weeks before the start of the study; or (c) with any condition (e.g., *communication*, sensory or motor deficits) that could significantly interfere with the neuropsychological assessment or to receive a cognitive intervention protocol of the trial will be excluded from the study. In addition, participants with contraindications to fMRI scans such as claustrophobia, presence of electronic implants (transient or definitive pacemaker, cochlear implant), or the presence of intracranial aneurysm clips will also be excluded. Finally, exclusion criteria relating to contraindications of neurostimulation will be applied, following the safety guidelines: (a) using psychoactive drugs, as stated in the recommendations; (b) having implanted metallic or electronic devices; (c) having a cardiac pacemaker, seizures, acute eczema under the electrodes; and (d) the presence of tumors, epilepsy, or substance abuse (Nitsche et al., 2003; Brunoni et al., 2011).

#### Patient Recruitment

A sample of volunteer participants will be recruited from neurorehabilitation clinics, community programs dementia treatment centers and the Aging Neuropsychology Service (SENE) of the Federal University of Paraíba. The clinical trial will also be promoted in websites, newspapers and radio. Furthermore, healthcare professionals from the aforementioned sites will promote the study among patients with probable AD. After signing the informed consent form, the data of patients interested in participating in the clinical trial will be analyzed, and the patients will be contacted for inclusion in the study, according to the eligibility criteria.

### Intervention

#### tDCS

Anodal tDCS (2 mA, 30 min) will be applied three times a week for 2 months (24 sessions). The TCT-Research neurostimulator, developed by TransCranial Technologies (Hong Kong, China) will be used. The rubber electrodes are involved in saline-soaked sponges and fixed with a headband. We will use a specific electrode (4 × 4 cm) to avoid covering of adjacent areas by the tDCS electrode (current density = 0.13 mA/cm^2^). After the electrodes placement, the stimulation duration, current intensity and both ramp up/ramp down times will be programmed. To avoid that stimulation fail, the impedance levels will be checked (constant under 5 kΩ). Proper and reliable stimulation requires good contact under the electrode area to a consistently conductivity through the circuit. In the case of stimulation failures, the problem can be solved by reapplying a saline solution or conductive gel on the holding bags, or by parting the hair beneath electrodes, as reported in previous studies. To ensure that any discomfort occurs, the participants will be accompanied during all experiment procedures (DaSilva et al., 2011; Thair et al., 2017).

**Figure 1:**
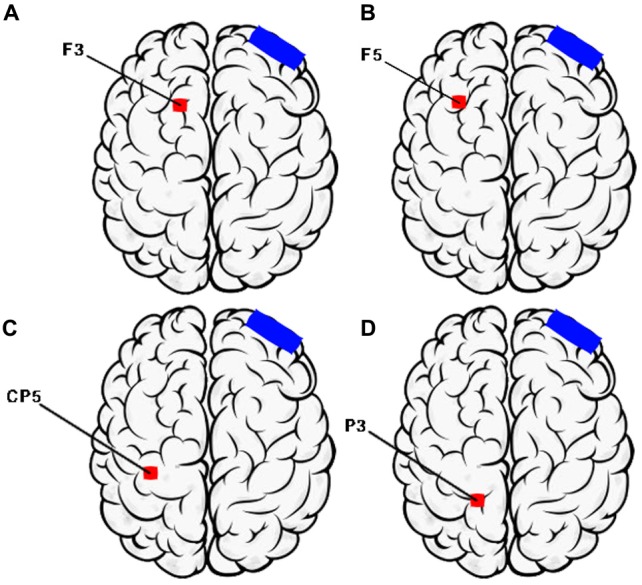
Montage of transcranial direct current stimulation (tDCS) sessions (Left Hemisphere). **(A)** F3—Dorsolateral prefrontal Cortex; **(B)** F5—Broca’s area; **(C)** CP5—Wernicke’s area; **(D)** P3—Somatosensory association cortex. Electrode position nomenclature of 10/20 System (Electroencephalography).

The active current will be applied to six cortical areas affected by AD. These sites are primary centers involved in the manifestation of clinical symptoms of the disease, including the left and right portions of the dorsolateral prefrontal cortex, related to short-term and long-term memory, judgment ability and executive functions; Broca’s area and Wernicke’s area, located in the temporal lobe, responsible for language; and the right and left somatosensory association cortex, in the parietal lobe, related to topographical and spatial orientation and praxis (Figure 1). The choice of these areas follows previously-tested neurostimulation protocols in patients with AD (Bentwitch et al., 2011; Rabey et al., 2013).

We will subdivide the neuromodulation protocol into A and B to stimulate the areas with the same number of sessions: (1) protocol A will involve stimulating Broca’s area (F5), Wernicke’s area (CP5), and the right dorsolateral prefrontal cortex (F4); and (2) protocol B will involve stimulating the left dorsolateral prefrontal cortex (F3) and the right and left portions of the parietal cortex (P4 and P5), according to the international 10–20 electrode nomenclature system. The protocols will be applied alternately; that is, protocol A will be applied in one session and protocol B in the next to stimulate the six cortical areas. The counterbalancing is justified to prevent higher current intensity applied to the patient’s scalp with the simultaneous stimulation of multiple areas, which could provoke painful stimuli and would not be in line with the technique guidelines (Furubayashi et al., 2008; Bikson et al., 2016).

The location of the protocol’s target regions to be applied on the day (either protocol A or B) will be performed before we begin the procedure. Thus, all regions are marked on the participant’s scalp in advance, avoiding delays during the treatment session. The instrument will cease after the first 10 min, and the electrodes will be positioned in the next region. As they are already marked, moving from one location to another will take less than 1 min (20–35 s estimate). Therefore, the treatment session will last at least 28 min, considering two brief stops for positioning the electrodes. We did not find evidence in the literature of interruptions of less than 1 min during electrode exchange or positioning, which could result in ablation or interference in the anode or current cathode effects.

Each aforementioned area will be stimulated for 10 min, totaling 30 min of treatment (three areas stimulated per session). The reference electrode 35 cm^2^ (5 × 7 cm) will be placed in the contralateral supraorbital area. The placebo stimulation protocol will be identical, although the device will cease to emit a current 30 s after starting the stimulation. Thus, the active current is simulated (slight tingling and itching sensation) for a short period, and the effects disappear shortly after the start of the stimulation (Nitsche et al., 2003; Gandiga et al., 2006).

An *fMRI* of all participants will be obtained before the experiment. The FSL software (FMRIB’s Software Library, University of Oxford, UK) will be used to transform coordinates for brain areas for each subject individually. The individual structural images will be initially converted into MNI coordinates (standard Montreal Neurological Institute) and then the MNI coordinates will be inversely transformed to the original imaging space. These values will be obtained by converting Talairach coordinates to the MNI space (Paus, 1996). These coordinates will be used to guide the frameless stereotaxy (Brainsight system, Rogue Research, Montreal, QC, Canada). The location of target regions will be performed by an experienced and trained professional. Procedures will be repeated at each treatment session for all participants involved.

#### Cognitive Intervention

The active protocol, involving 72 cognitive activities, was developed by neuropsychologists specialized in the non-pharmacological treatment of AD and will be applied by professionals trained in the management of this cognitive intervention program. This program is supported by similar studies of cognitive intervention in this population (Spector et al., 2001; Fernández-Calvo et al., 2010, 2015).

The cognitive activities with correspondence to the stimulated areas were developed using validated paradigms: (a) syntax and grammar tasks for Broca’s area and comprehension of lexical meaning and categorization for Wernicke’s area; (b) verbal and spatial working memory tasks for the dorsolateral prefrontal cortex; and (c) Spatial attention and perceptual–motor tasks (shapes and letters) for the somatosensory association cortex (e.g., Dobbs and Rule, 1989; Gabrieli et al., 1996; Buck et al., 1997; Thompson-Schill et al., 1997; Grossman and Rhee, 2001; Grossman et al., 2002; Love and Oster, 2002; Hao et al., 2005; Sparing et al., 2008; Bellgowan et al., 2009; Hamidi et al., 2009; Nyberg et al., 2009; Belleville et al., 2011; Hampstead et al., 2011). Most of these paradigms were applied in studies with AD patients or with neurostimulation techniques. Similar tasks based on the same paradigms were tested in studies that combined neuromodulation and cognitive intervention for people with AD (e.g., Bentwitch et al., 2011). However, some of these tasks have been adapted from the original paradigms to be used in pencil-and-paper activities. Some of the cognitive activities that will be used with the participants of the clinical trial are described below:

- ***Cognitive activities associated with Broca’s area***. This set of tasks tries to activate verbal language production through stimulating syntactic and grammatical language processing:
  - **Repeating words, pseudowords and phrases**. The participant should repeat the words (concrete nouns that vary in size and frequency), pseudowords and phrases created from the previous stimuli, which are presented orally.
  - **Naming**. The participant is instructed to retrieve the name of the displayed object. If the participant does not name it (e.g., bed), then both semantic (e.g., a piece of furniture) and phonological tips will be provided (e.g., begins with ca).
  - **Description-Narrative by confrontation**. This task consists of stimulating expressive conversation language based on visual stimuli. The participant will be shown a thematic slide about a situation and the situation should describe what they see, producing a story. It is about creating phrases and producing descriptive narratives and not simply naming objects in the image. For example, from the thematic sheet 1: “The city” can be elaborated into a story like the following: The girl went out to the street … She was going to buy … She arrived at the traffic light … She crossed the street without looking at the traffic lights … The driver … had to brake abruptly … The blind man was in the same …”
- ***Cognitive activities associated with Wernicke’s area***. This type of activity focuses on triggering language comprehension using the lexical/semantic system:
  - **Sentence comprehension**. This task consists of silently reading incomplete sentences and the participant should write the word that completes the sentence (e.g., dogs have four‥.). If the participant does not give an answer, four words (e.g., fingers, legs, legs, eyes) are shown and the participant must select the one that best serves to complete the sentence. One variant of this task is understanding contextualized phrases, which consists of understanding phrases contextualized by images. The participant should read a sentence, analyze the images and mark the one that best represents the read sentence (e.g., the phrase “The driver drives the bus” appears next to four pictures, in which only one corresponds to the sentence read).
  - **Association of nouns and adjectives**. In this task, different nouns and adjectives are presented and the participant should join the most appropriate adjective for each word with the arrows (e.g., teeth, green, hair, fair, litigation, maturity, grassland, white, fruit, gray-haired).
  - **Grammatical class change**. This task consists of exchanging nouns for verbs and verbs for nouns. In changing the condition from noun to verb, participants are instructed to read the noun and retrieve the verb (e.g., Forgiveness = forgive). In the case of not issuing a response, a list of verbs will be shown in which the participants will retrieve the verb corresponding to the noun. In the condition of verb for noun, participants should read the verb and retrieve the corresponding noun (e.g., amar = lover).
- ***Cognitive activities associated with left or right dorsolateral prefrontal cortex***. These activities are focused on promoting manipulation of verbal and spatial knowledge, associative processes and information selection:
  - **Object-location memory**. This task includes the coding and recognition stages of alternating stimuli or sites. In the coding and recognition phases, different sequences of six stimuli (shapes, colors, faces and letters) are presented. The stimulus memory task requires the subsequent recognition of target stimuli regardless of location, while the spatial task requires the coding and subsequent recognition of the location at which the stimuli were presented.
  - **Delayed match-to-sample tasks**. This type of activity requires visual pairing with the delayed model. The procedure consists of presenting a stimulus that the participant must remember (sample). Then, the stimulus is withdrawn and the participant must identify the stimulus that exactly corresponds to the sample (comparison stimulus) among a set of stimuli. The target and comparison stimuli can be simple (pictures of shapes, colors, letters, faces and objects) or complex (abstract). Thus, different variations of this task were created.
  - **Selection of semantic knowledge**. This task consists of classifying designs of common objects while taking into account different selection conditions such as a specific attribute of the object’s representation (large, small, light, heavy, artificial, natural, expensive, cheap) or according to the object’s name (spider, hammer). As an example of the first condition, a drawing of a car is presented together with the word car, whereas in the second condition the word spider is next to the drawing of a swan. In both activities, the participants should determine whether this classification is correct or not.
  - **Associative face memory**. This task requires encoding and recognizing associations between faces and names. In the first condition, subjects visualize paired faces with an unusual name. After the participant observes four pairs of faces with their names, one face and four name options are displayed.
  - **N-back task**. This task requires the participant to decide whether each heard or seen sequential stimulus matches what was heard or seen *n* items before. The stimuli can be letters, numbers, words (auditory condition) or photographs of objects (animals, fruits, tools, household utensils), and three conditions are used for each type of stimulus (*n* = 0, *n* = 1 and *n* = 2). For example, in condition *n* = 0, the participant must repeat the letter or (number) immediately after listening to it [C (“C”), F (“F”). T (“T”), S (“S”), B (“B”)]; in *n* = 1, the participant should say the letter they heard immediately before [C (“none”), F (“C”). T (“F”), S (“T”), B (“S”)]; and in *n* = 2, the participant has to say the letter they heard two times before [C (“nothing”), F (“nothing”), T (“C”), S (“F”), B (“S”)].
  - **Encoding and retrieving word meaning**. This task requires remembering words using a semantic coding strategy. The implemented stimuli are concrete (e.g., cat, notebook) and abstract nouns (e.g., beauty, fear) and the participant must judge whether each word refers to something abstract or concrete. Once the judgment of each word presented has been completed, the participant is asked to remember the most words they can.
- ***Cognitive activities associated with somatosensory association cortex***. These exercises aim to stimulate cognitive components related to perception (e.g., recognition of simple and complex objects or spatial relationships between them), spatial attention (e.g., visual scanning and tracking), visual episodic memory (e.g., task retrieval), visual working memory (task recognition) and motor skills or spatial reasoning:
  - **Match-to-sample task**. This activity requires simultaneous visual matching capability. It consists of simultaneously displaying a simple or complex visual stimulus (sample) and four comparison stimuli. The participant should select the stimulus that exactly corresponds to the sample. The target and comparison stimuli can be simple (line drawings or pictures of shapes, letters, faces, or objects) or complex (abstract). Thus, different variations of this task were created.
  - **Visual search**. This type of activity requires the selection of relevant stimuli (target) and ignoring irrelevant (distracting) stimuli. The task requires the participant to detect the predefined target stimuli in a matrix in the midst of a series of distracting items. Different versions of this task were created using different types of stimuli (pictures and line drawings of shapes, letters, or objects). Two classes of stimuli are present in some situations in the matrix, requiring them to link characteristics to perform the activity. For example, the target is a vertical red bar and the distractors are vertical green bars and red horizontal bars.
  - **Overlapping figures**. This task requires identifying overlapping stimuli (shapes, letters and line drawings) with different levels of noise.
  - **Visual Tracking**. This task requires following a curved and irregular line with their eyes from one attached point to another attached point associated with numbers or letters. For example, the participant must choose a letter (or number) and follow the connecting line to its destination number (or letter) using only their eyes, without paying attention to the lines that they cross.

The 72 cognitive activities distributed by the stimulated areas each week were organized in a book, which includes the description (objective and procedure) and the material needed for each activity. All occupational therapists will be trained to apply each cognitive activity in the same way before beginning the clinical trial. Monitoring sessions will be held monthly to solve any problems in the intervention.

All activities will be applied simultaneously with neurostimulation, that is, the occupational therapist will perform activities involving operational memory while the operational memory area is stimulated with tDCS; cognitive intervention activities will focus on language when stimulating the language cortical area, and so on so that neurostimulation is related to the cognitive task tested. The 10-min time per region will also apply to the cognitive intervention protocol, since tasks will be scheduled to last up to this time. Thus, when the device ceases to emit the current, the cognitive task will not be “interrupted”, but rather finalized, because we expect to perform three different types of exercises over 30 min, each lasting 10 min. The pilot studies will allow us to calibrate a number of cognitive exercises necessary for each of these activities within each intervention session. Since the electrode change for a new region will be performed in less than 1 min, we think the interruption effect is minimal. Nevertheless, in order to minimize possible effects through the cognitive activity intervention, participants will be informed of the three groups of activities that will be performed at the beginning of each session, and the duration of each activity. However, even though the patient can perform them in a slower or faster time than this, we expect that this variation should be minimal because the total time is equivalent to the defined time in the protocol.

Regarding the placebo protocol of cognitive intervention, participants will watch short videos while receiving tDCS. The videos of the sham cognitive stimulation will also be chosen based on this time limit of 10 min. The researcher responsible for the cognitive intervention will stand next to the participant during the session and make comments unrelated to the treatment, including asking what the patient found from the video, whether it was pleasurable, and whether they are feeling comfortable.

### Outcome Measures

All outcome measures for AD patients will be done in 60-min session, pre and post intervention phase. The Reliable Change Index (RCI) will be used to minimize the practical effects, as described in the “Proposed Analysis” section.

#### Primary Outcome

The AD Assessment Scale, cognitive subscale (ADAS-Cog; Rosen et al., 1984), will be used to analyze the primary outcome. The ADAS-Cog consists of an 11-item cognitive subscale related to memory, orientation, visuo-spatial ability, language and ideational praxis. The main cognitive domains assess memory, language, praxis and understanding. The instrument has inter-rater reliability values ranging from 0.947 to 0.99 and test-retest values ranging from 0.59 to 0.92, and it is considered reliable for application to patients with AD. Scores range from 0 to 70, with higher scores reflecting more cognitive impairment.

#### Secondary Outcomes

##### The Neuropsychiatric Inventory Questionnaire (NPI-Q; Kaufer et al., 2000)

This instrument is completed by caregivers and provides information regarding 12 typical behavioral and psychological symptoms that may occur in patients with dementia. In this study, we used a severity rating (1 = mild, 2 = moderate, 3 = severe) for identified symptoms, deriving a severity score (range 0–36), with zero indicating no neuropsychiatric symptoms. The reliability and validity of this modified NPI score have previously been established.

##### The Cornell Scale for Depression in Dementia (CSDD; Alexopoulos et al., 1988)

This instrument is a 19-item clinician-administered instrument that uses patient and caregiver information to assess symptoms of affective disturbance in patients with dementia. It assesses mood-related signs, behavioral disturbances, physical signs, cyclic functions and ideational disturbances. Each item of the Cornell Scale for Depression in Dementia (CSDD) is evaluated on a three-point scale (0–2) that reflects symptom severity. Hence, the total score ranges from 0 to 38, with higher scores depicting greater depression. The reliability and validity of this CSDD score have been previously established.

##### The Disability Assessment for Dementia (DAD; Gélinas et al., 1999)

This instrument is an informant-based scale composed of 17 items pertaining to basic ADLs (hygiene, dressing, continence and eating) and 23 items pertaining to instrumental ADLs (meal preparation, telephoning, going on an outing, finance and correspondence, medications and leisure and housework). Each item is evaluated for initiation, planning and effective execution. To avoid bias toward certain activities (e.g., meal preparation), nonapplicable questions are omitted from the final score, and the score is reported as a percentage. The maximum total score is 100, with lower Disability Assessment for Dementia (DAD) scores denoting greater impairment. Basic ADL and instrumental ADL subscores are included in the analysis, in addition to total DAD scores (Bahia et al., 2010).

##### The Zarit Burden Interview (Zarit et al., 1985)

This instrument (22 items) is used to measure caregivers’ perceived burden in diverse domains (Zarit et al., 1985). The scale was validated for the Brazilian population (Taub et al., 2004), with acceptable internal reliability (Cronbach’s alpha), ranging from 0.77 to 0.88. The score responses range from 0 (never) to 4 (almost always) to prevent discrepancies with the original scale scores. Total scores range between 0 and 88 points, with higher scores reflecting higher caregiver burden.

Given the possibility of biases associated with this procedure, we perform the neuropsychological assessments on two consecutive days, respecting the limits of patients and their caregivers and offering them breaks for rest and hydration, whenever necessary.

##### Brain-Derived Neurotrophic Factor (BDNF)

Serum brain-derived neurotrophic factor (BDNF) levels will be measured with the BDNF Emax Immunoassay system kit (Promega, Madison, WI, USA), according to the manufacturer’s instructions. Blood samples will be collected and allowed to coagulate for 1 h at room temperature, followed by 1 h at 4°C. Serum will be separated by centrifugation at 2000 g for 10 min at 4°C, put in an aliquot and stored at −80°C until used in 0.2 ml tube strips.

### Ethics, Participant Consent and Study Registration

This study is being carried out in accordance with the recommendations of National Health Council with written informed consent from all subjects. All subjects will provide written informed consent in accordance with the Declaration of Helsinki. The protocol was approved by the Institutional Ethics Committee on Human Research. In case of inability to understand the instructions or due to the presence of anosognosia, both the participant and caregiver will sign the informed consent form. The study was approved by the *Brazil’s National Committee for Ethics in Research* (CAAE: 44388015.7.0000.5188) and publicly registered in the ClinicalTrials.gov database (NCT02772185). The trial protocol follows the SPIRIT (Standard Protocol Items: Recommendations for Interventional Trials) guidance for protocol reporting (Chan et al., 2013).

### Sample Size

The sample size was estimated based on previous studies that used paired tDCS groups in treating AD (Ferrucci et al., 2008; Boggio et al., 2012). The power calculations determining the number of participants in each group were made in relation to the expected change in ADAS-Cog because this instrument is commonly used in studies involving cognitive evaluation in these patients (Vellas et al., 2007; Balietti et al., 2017; Xiao et al., 2017).

The expected significant improvement is reaching 3.76 points in the mean, with an standard deviation (SD) of 1.32 points, according to Rabey et al. (2013). Therefore, a calculation considering the level of *p* < 0.05 and 90% power as significant suggests that at least 10 patients would be necessary in each group to detect whether the difference found matches the effect of the active or simulated treatment. Considering the application of tDCS in multiple areas, stimulation time and the possibility of sample losses throughout the study (dropout, inability to continue treatment, death), 25 patients per group will be included, totaling 100 participants.

#### Proposed Analysis

Intent-to-treat analysis will be used; that is, all participants will be included in the analysis, including those who drop out before completing the treatment. However, high or different rates of dropouts between the treatment arms may lead to biases. Thus, a sensitivity analysis will be performed with different assignment procedures to assess the value of the data. The best strategy resulting from the following methods will be chosen: last observation carried forward, complete case analysis, likelihood-based methods, or multiple imputation.

Descriptive statistics will be used to describe clinical and sociodemographic characteristics and the primarily and secondary outcomes of each group at the baseline. The groups will be compared by analysis of variance (ANOVA), for continuous variables, or the chi-square test, for categorical variables.

The primary outcome will be examined by split-plot ANOVA with one dependent variable (ADAS-Cog score) and two independent variables, namely, one intra-group variable and one inter-group variable. The within-subject variable will include time (baseline, post-treatment and 12-month follow-up), and the effects of the treatment condition (active or placebo tDCS; active or placebo cognitive intervention) will be examined as a between-groups variable. *Post hoc* comparisons using Bonferroni correction will be performed where appropriate. Finally, the effect sizes regarding inter-group comparisons will be calculated using the eta squared (*η*^2^), and effect sizes regarding intra-group comparisons will be calculated by means of Cohen’s d using Hedges’ correction (Fritz et al., 2012), adopting the significance level of *p* < 0.05. For all variables of the secondary outcomes, the same analysis strategy will be used. Non-parametric tests will be performed if some of the outcomes do not meet the conditions necessary for parametric analysis.

Considering the different evaluations with the same cognitive tests throughout the clinical trial, the improvement in the performance of these tests may result from practice and not from the intervention applied (Chelune, 2010; Goldberg et al., 2015). A RCI will be used; it serves to assess whether the changes identified comparing pre- and post-intervention data can be attributed to the procedures used or are mere oscillations, artifacts, or measurement errors (Jacobson and Truax, 1991) resulting from repeated practice effects (Chelune et al., 1993). The RCI, correcting for practice effects and using the Iverson’s standard error of the difference will be calculated based on the Duff’s (2012) formulae.

The follow-up analysis will calculate the primary outcome (i.e., a decline in cognitive state). As proposed in Phase 1, the same cutoff will be used to classify clinical improvement (3.76 points in the mean, with an SD of 1.33 points in the ADAS-cog). The primary outcome measure will be calculated considering the differences between clinical improvement and clinical relapse (or censored if relapse did not occur). Kaplan-Meier survival analysis will be used to calculate this outcome. To investigate interactions of risk factors, the following variables will be observed: demographics characteristics (age, gender, level of education), clinical condition (duration of illness, comorbidities and neuropsychiatric states) and “methodological” variables (Phase 1 group and crossover). The hazard ratio will be estimated using the Cox proportional hazards (to each predictor variable or covariate)”

Regarding safety, adverse effects will be analyzed in terms of the ratio in each group and period (before and after treatment) and analyzed using Fisher’s exact test. Multivariate regression will be used to identify predictors of response. The outcome variables will be tDCS and cognitive intervention, and the predictors will be age, gender, comorbidities and use of medication.

The effectiveness of the blinding will be analyzed by using the chi-square test, comparing the ratio between errors and correct responses. Furthermore, these tests will be compared between patients with clinical response and those who failed to improve.

Multivariate imputation by chained equations (MICE, the R Foundation for Statistical Computing, Vienna, Austria; van Buuren and Groothuis-Oudshoon, 2011) and the Statistical Package for Social Sciences (SPSS) version 21 (IBM Corp. Released, 2011, IBM^®^, SPSS Statistics version 21) software will be used.

## Stepwise Procedures

**Figure 2:**
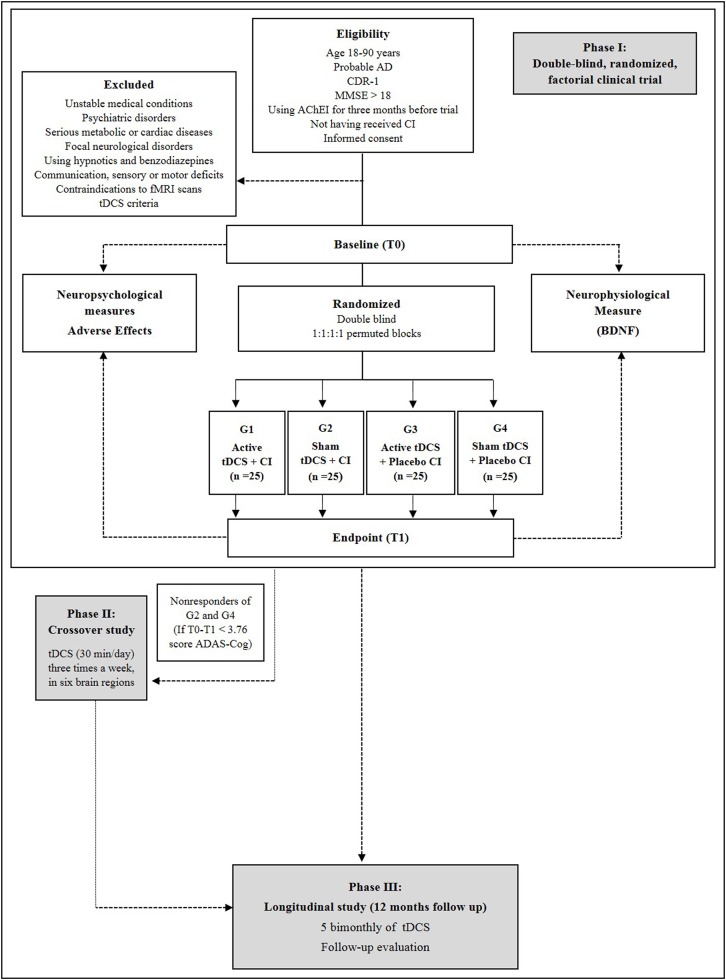
Flowchart of phases I (double-blind factorial clinical trial), II (crossover) and III (follow-up) of the Neurostimulation combined with Cognitive Intervention in Alzheimer’s disease (AD) Study (NeuroAD). T0: Baseline, pre-intervention measurement. T1–T8: intervention, tDCS and cognitive treatment. T9: endpoint, post-intervention measurement.

Figure 2 shows the stages of NeuroAD.

The phase I is a double-blind, randomized, factorial clinical trial in which tDCS will be applied in parallel to cognitive intervention in AD patients. This stage includes two measurement time points: baseline (week 0) and post-treatment (week 8). The data collection sessions will be performed by treatment-blinded investigators including the primary and secondary outcomes assessments. The pre-measurement consists of a screening and measurement part. The screening part contains data of the DSM-IV-TR, NINCDS-ADRDA criteria, CDR, MMSE and health questionnaire (including questions about disease duration, comorbidities and use of medication). Adverse effects will be assessed at each session, wherein the tDCS rater will ask the patients whether they feel any discomfort or pain and whether this could be related to tDCS.

Phase II is an open-label, crossover phase in which non-responders from groups (2) and (4) (i.e., those receiving sham tDCS) receive a course of active tDCS using the same parameters and montage of phase I, that is, a direct current of 2 mA (current density = 0.13 mA/cm^2^) for 30 min/day for 24 days, in respective six brain regions.

Phase III is the longitudinal study of NeuroAD in which all active-tDCS responders—that is, responders from groups (1) and (3) from phase I and responders from phase II will be enrolled in a bi-monthly 1-year follow-up. Thus, two months after the last session, the participants will receive ten consecutive tDCS sessions (from Monday to Friday), which will be repeated every other month, until completing a one-year period (totaling 70 follow-up sessions). The tDCS parameters will be the same as those applied during the period between baseline and post-treatment, and the same cortical areas will be stimulated.

The expected significant improvement is reaching 3.76 points in the mean ADAS-Cog, with a SD of 1.32 points, according to Rabey et al. (2013). Thus, we consider the difference of at least three points in the ADAS-Cog and in the pre- and post-treatment measures to be clinical improvement. All participants with clinical improvement will be considered as respondents.

In all steps, the primary and secondary outcomes assessments will be repeated. The pre-intervention measurement is planned to be performed in the week before the intervention starts and the post-intervention measurement is planned for the first week after the intervention.

## Anticipated Results

Considering there is a serious lack of factorial designs for clinical trials in non-invasive cerebral stimulation for AD patients, we aim to ameliorate that situation by observing the effect size and clinical effectiveness of each procedure, not only comparing this intervention against a sham (which could prove the efficacy, but not the clinical effectiveness), but also against a cognitive intervention. Further, we hope to broaden the treatment scope, promoting improvement in cognitive and functional aspects by stimulating different cortical regions, in addition to memory. Therefore, our study may generate key results for clinical practice and patient management throughout the disease.

Then, to the best of our knowledge, our study may be the first to apply a factorial design in non-invasive cerebral stimulation for AD patients, and not only comparing this intervention against a sham (which could prove the efficacy, but not the clinical effectiveness), but also against a cognitive intervention. Next, this factorial design will be able to observe the effect size and clinical effectiveness of each procedure, and will also be able to elucidate whether it would actually be feasible to apply the therapies alone or in combination. Further, we hope to broaden the treatment scope, promoting improvement in cognitive and functional aspects by stimulating different cortical regions, in addition to memory. Therefore, our study may generate key results for clinical practice and patient management throughout the disease.

An additional relevant issue is the longer-lasting effects. Evidence shows that multisession protocols promote neural plasticity and after-effects in AD and other conditions (Fregni et al., 2015; Bystad et al., 2016b). Although a case study that evaluated the effects of tDCS up to 8 months has been reported in the literature (Bystad et al., 2017), we hope to find that bimonthly stimulation sessions amplify the produced effects. Likewise, the use of long-term physiological markers will allow us to know what kind of non-pharmacological therapies produce greater neuroplasticity effects in AD patients, which are responsible for reaching and maintaining better long-term overall performance. The implementation of more effective procedures of plasticity induction in clinical settings is essential—in fact, as a maintenance treatment for bimonthly sessions, tDCS can offer the advantage of a cost-effective approach (reducing missed visits) and a less expensive alternative compared to daily sessions in the long-term. In this way, our findings may also help improve the recommendation management of AD.

Fundamentally, our study presents some methodological limitations that should be discussed, including adherence. The participants must come to our laboratory to receive 24 sessions three times a week. This may result in absences due to transportation problems or personal difficulties. However, different strategies will be adopted to minimize these problems, such as flexible hours and frequent phone calls, encouraging and reinforcing the importance of treatment. Also, we will set a limit of missed appointments so that the treatment process is less rigid, and the patient can resume the missed sessions without compromising the treatment follow-up if necessary. Second, applying the neuropsychological tests could elicit a learning effect. The practice-adjusted RCI of the different cognitive tests used in the clinical trial will be used to minimize this limitation (Goldberg et al., 2015), as described in the statistical analysis. Although some tests may have increased susceptibility to practice effects (Calamia et al., 2012), patients with AD show no significant effects in practice regarding repeated administrations of different cognitive tests after a test-retest interval (Cooper et al., 2004; Foley et al., 2015). A third consideration is the inclusion of only mild AD patients, thereby limiting the generalization to other phases of the disease. Considering cognitive and functional differences between the different stages of AD, we chose to evaluate patients at only one stage of the disease for improved data control. However, this does not preclude future studies comparing patients at different stages of the disease to specifically clarify the best dosage, procedure and number of tDCS sessions and cognitive intervention, considering the clinical symptoms and disease progression of the patients. Finally, although our proposition is to stimulate different regions affected by AD, we cannot assure that there will be no cumulative effects between them. In view of intersubject and intrasubject variations, many studies claim to have the focal power on tDCS, so we cannot rule out the possibility of effect propagation from one region to another (Boros et al., 2008; Cunningham et al., 2015). As our study does not aim to evaluate or compare the efficacy of tDCS at each site separately, cumulative effects from synaptic connections between the involved regions may result in improved cognitive and functional performance, and not cause harm to patients. Nevertheless, it is important to emphasize that we will use several strategies to control bias, such as reduced electrode size, use of neuronavigation to localize target regions in each patient, and the use of standardized coordinates from previous studies for localizing target regions.

In general, we hope that our results can elucidate the relevance of such potential limitations. Additionally, we consider that the current protocol together with our results will have important implications for AD treatment, both theoretically in providing support for new trials and for clinical management, thus providing evidence applicable to the routine care of these patients.

## Author Contributions

SA and BF-C developed and drafted this study protocol in consultation with EO, NA, MQCG and MR. RN and IC provided inputs to the design of the customized group neurostimulation and cognitive program intervention. AS, CM, DS, ES, EF, ER and GL prepared the human research ethics. JC, JS, MT, MO, ML, NL, PR, RF and RC were involved in the overall research design and selection of outcome measures. RA, RS, PI, TP and WM were involved in the design and facilitation of the group program intervention. TF and VP were involved in the mixed-methods research methodologies, outcome measures and structured interview questions. All authors listed have made a substantial, direct and intellectual contribution to the work and approved it for publication.

## Conflict of Interest Statement

The authors declare that the research was conducted in the absence of any commercial or financial relationships that could be construed as a potential conflict of interest.

## Footnotes

^1^www.random.org
